# Supplementary material for: Mannose receptor is an HIV restriction factor counteracted by Vpr in macrophages
Source: eLife. 2020 Mar 2;9:e51035. doi: 10.7554/eLife.51035 (PMC7051176; doi:10.7554/eLife.51035)
Supplement: Supplementary file 1. [file elife-51035-supp1.docx]

| **Reagent type (species)** | **Designation** | **Source or reference** | **Identifiers** | **Additional**  **Information** |
| --- | --- | --- | --- | --- |
| Recombinant DNA reagent | p89.6 | Collman, Balliet et al. 1992 PMID: 1433527 | NIH AIDS Reagent Program 3552 |  |
| Recombinant DNA reagent | p89.6 vpr-null | Mashiba, Collins et al. 2014; PMID 25464830 |  |  |
| Recombinant DNA reagent | p89.6 nef-null | Carter, Onafuwa-Nuga et al. 2010; PMID 20208541 |  |  |
| Recombinant DNA reagent | p89.6 vpr-nef-null | this paper |  | Produces HIV 89.6 vpr-nef-null double mutant |
| Recombinant DNA reagent | p89.6 env N230D N339E | this paper |  | Produces HIV 89.6 env N230D N339E mutant |
| Recombinant DNA reagent | p89.6 env N230D N339E vpr-null | this paper |  | Produces HIV 89.6 env N230D N339E vpr-null mutant |
| Recombinant DNA reagent | p89.6 env N230D N339E vpr-nef-null | this paper |  | Produces HIV 89.6 env N230D N339E vpr-nef-null mutant |
| Recombinant DNA reagent | pNL4-3 | Adachi, Gendelman et al. 1986; PMID 3016298 | NIH AIDS Reagent Program 114 |  |
| Recombinant DNA reagent | pNL4-3 env^YU2^ | this paper |  | Produces HIV NL4-3 env^YU2^ chimera |
| Recombinant DNA reagent | pNL4-3 env^YU2^ vpr-null | this paper |  | Produces HIV NL4-3 env^YU2^ vpr-null chimera |
| Recombinant DNA reagent | pHCMV-G | ATCC | 75497 | Expresses VSV-G |
| Recombinant DNA reagent | pCMV-HIV-1 | Gasmi, Glynn et al. 1999; PMID 9971760 |  | Expresses HIV structural proteins |
| Recombinant DNA reagent | pNL4-3 ∆GPE-GFP | McNamara, Ganesh et al. 2012; PMID 22718820 |  |  |
| Recombinant DNA reagent | pNL4-3 ∆GPE-GFP vpr-null | this paper |  | Produces NL4-3 ∆GPE vpr-null |
| Recombinant DNA reagent | pNL4-3 ∆GPE-GFP vpr-Q65R | this paper |  | Produces NL4-3 ∆GPE vpr-Q65R |
| Recombinant DNA reagent | pNL4-3 ∆GPE-GFP nef-null | this paper |  | Produces NL4-3 ∆GPE nef-null |
| Recombinant DNA reagent | pNL4-3 ∆GPE-GFP vpr-nef-null | this paper |  | Produces NL4-3 ∆GPE vpr-nef-null |
| Recombinant DNA reagent | pYU2 | Li, Kappes et al. 1991; PMID 1830110 | NIH AIDS Reagent Program 1350 |  |
| Recombinant DNA reagent | pYU2 vpr-null | this paper |  | Produces YU-2 vpr-null |
| Recombinant DNA reagent | pREJO.c/2864 | Ochsenbauer, Edmonds et al. 2012; PMID 22190722 | NIH AIDS Reagent Program 11746 |  |
| Recombinant DNA reagent | pREJO.c/2864 vpr-null | this paper |  | Produces REJO vpr-null |
| Recombinant DNA reagent | pSIV3+ | Pertel, Reinhard et al. 2011; PMID 21696578 |  |  |
| Recombinant DNA reagent | pSIV3+ vpr-null | this paper |  | Produces SIV3+ vpr-null |
| Recombinant DNA reagent | pSPAX2 | Pertel, Reinhard et al. 2011; PMID 21696578 |  |  |
| Recombinant DNA reagent | pAPM-1221 | Pertel, Reinhard et al. 2011; PMID 21696578 |  | Silences luciferase mRNA |
| Recombinant DNA reagent | pAPM-MRC1-C | this paper |  | Silences MR mRNA |
| Recombinant DNA reagent | pMD2.G | Pertel, Reinhard et al. 2011; PMID 21696578 |  | Expresses VSV-G |
| Recombinant DNA reagent | pYU2 env | Sullivan, Sun et al. 1995; PMID 7769703 |  |  |
| Recombinant DNA reagent | pCDNA3.hMR | Liu, Liu et al. 2004; PMID 15047828 |  | Expresses MR |
| Recombinant DNA reagent | pPROA-3FLAG-UNG2-EYFP | Akbari, Solvang-Garten et al. 2010; PMID 20466601 |  |  |
| Recombinant DNA reagent | pMSCV IRES-GFP | Van Parijs, Refaeli et al. 1999; PMID 10514006 |  |  |
| Recombinant DNA reagent | pMSCV 3xFLAG UNG2 IRES-GFP | this paper |  | Expresses 3x FLAG-tagged UNG2 |
| Recombinant DNA reagent | pUC19 | Norrander et al. 1983; PMID 6323249 |  |  |
| Chemical compound, drug | Ficoll-Paque Plus | GE Healthcare | 17-1440-02 |  |
| Chemical compound, drug | rhM-CSF | R&D Systems | 216-MC-025/CF |  |
| Chemical compound, drug | rhGM-CSF | R&D Systems | 215-GM-050 |  |
| Chemical compound, drug | IL-2 | R&D Systems | 202-IL-010 |  |
| Chemical compound, drug | phytohaemagglutinin-L | Calbiohem | 431784 |  |
| Chemical compound, drug | Enzyme-free cell dissociation buffer, HBSS-based | ThermoFisher | 13150016 |  |
| Chemical compound, drug | Blue loading buffer | Cell Signaling Technology | 7722 |  |
| Chemical compound, drug | AMD3100 | Hendrix, Flexner et al. 2000; PMID 10817726 | NIH AIDS Reagent Program 8128 |  |
| Chemical compound, drug | Maraviroc | Emmelkamp and Rockstroh 2007; PMID 17933722 | NIH AIDS Reagent Program 11580 |  |
| Chemical compound, drug | streptavidin-HRP | Fitzgerald | 65R-S104PHRP |  |
| Chemical compound, drug | 3,3',5,5'-tetramethylbenzidine | Sigma | T8665-IL |  |
| Chemical compound, drug | Gag p24 standard | ViroGen | 00177-V |  |
| Chemical compound, drug | Protein G Column | GE Healthcare | 45-000-054 |  |
| Commercial assay, kit | Q5 site-directed mutagenesis kit | New England Biolabs | E0554S |  |
| Commercial assay, kit | EasySep™ Human CD14 Positive Selection Kit II | Stemcell Technologies | 17858 |  |
| Commercial assay, kit | CD8 Dynabeads | ThermoFisher | 11147D |  |
| Commercial assay, kit | RNeasy micro RNA isolation kit | Qiagen | 74004 |  |
| Commercial assay, kit | qScript cDNA Supermix | Quantabio | 95048 |  |
| Commercial assay, kit | TaqMan Gene Expression Master Mix | ThermoFisher | 4369016 |  |
| Commercial assay, kit | EZ-link Micro Sulfo-NHS-Biotinylation kit | ThermoFisher | PI-21925 |  |
| Sequence-based reagent | 896 dNef-F | this paper | PCR primer | CACCATTATCGTTTCAGACCCT |
| Sequence-based reagent | 896 dNef-R | this paper | PCR primer | TCTCGAGTTTAAACTTATAGCAAAGCCCTTTCCA |
| Sequence-based reagent | NL43 vprQ65R-Forward | this paper | PCR primer | AGAATTCTGCGACAACTGCTG |
| Sequence-based reagent | NL43 vprQ65R-Reverse | this paper | PCR primer | TATTATGGCTTCCACTCC |
| Sequence-based reagent | 3xFLAG UNG2 F | this paper | PCR primer | CTAGCTCGAGACCATGGACTACAAAGACCATGAC |
| Sequence-based reagent | 3xFLAG UNG2 R | this paper | PCR primer | GTTAACTCACAGCTCCTTCCAGTCAATGGGCTT |
| Sequence-based reagent | GeneExpression assay for ACTB | ThermoFisher | Hs99999903 |  |
| Sequence-based reagent | GeneExpression assay for MRC1 | ThermoFisher | Hs00267207 |  |
| Sequence-based reagent | GeneExpression assay for POL2A | ThermoFisher | Hs02786624 |  |
| Sequence-based reagent | GeneExpression assay for GAPDH | ThermoFisher | Hs00172187 |  |
| Sequence-based reagent | APM-MRC1-C Forward oligo | Sigma | DNA oligo | TCGAGAAGGTATATTGCTGTTGACAGTGAGCGAGTAACTTGACTGATAATCAATTAGTGAAGCCACAGATGTAATTGATTATCAGTCAAGTTACTTGCCTACTGCCTCGG |
| Sequence-based reagent | APM-MRC1-C Reverse oligo | Sigma | DNA oligo | AATTCCGAGGCAGTAGGCAAGTAACTTGACTGATAATCAATTACATCTGTGGCTTCACTAATTGATTATCAGTCAAGTTACTCGCTCACTGTCAACAGCAATATACCTTC |
| Biological sample (Homo sapiens) | Buffy coats/LeukoPaks | New York Blood Center |  | Buffy coats made from whole blood |
| Biological sample (adenovirus) | Adeno-nef | Leonard, Pilzen et al. 2011; PMID 21543478 |  |  |
| Cell line (homo sapiens) | HEK293T | ATCC | CRL-3216 |  |
| Cell line (mus musculus) | anti-gp41 hybridoma CHESSIE-8 | Abacioglu, Fouts et al. 1994; PMID 8068416 | NIH AIDS Reagent Program 526 | Purified ab used for WB (2µg/mL) |
| Cell line (mus musculus) | anti-p24 hybridoma 183-H12-5C | NIH AIDS Reagent Program | 1513 | Purified ab used for ELISA (1µg/mL) |
| Cell line (mus musculus) | anti-p24 hybridoma 31-90-25 | ATCC (discontinued) | HB-9725 | Purified ab used for ELISA (0.5µg/mL) |
| antibody | anti-mannose receptor-PE (mouse monoclonal) | Becton Dickinson | clone 19.2 cat# 555954 | FC (1µL per test) |
| antibody | anti-Gag CA p24-PE (mouse monoclonal) | Beckman Coulter | clone KC57 cat# 6604667 | FC (0.25µL per test) |
| antibody | anti-Gag CA p24-FITC (mouse monoclonal) | Beckman Coulter | clone KC57 cat# 6604665 | FC (0.25µL per test) |
| antibody | anti-FLAG (mouse monoclonal) | Sigma | clone M2 cat# F3165 | FC (1µL per test), WB (1:1000) |
| antibody | anti-CD4-APC (mouse monoclonal) | ThermoFisher | clone OKT4 cat# 17-0048-42 | FC (1µL per test) |
| antibody | anti-CD3-PacBlue (mouse monoclonal) | BioLegend | clone OKT3 cat# 317313 | FC (1µL per test) |
| antibody | anti-CD14-APC (mouse monoclonal) | BioLegend | clone HCD14 cat# 325608 | FC (1µL per test) |
| antibody | anti-mannose receptor (rabbit polyclonal) | Abcam | ab64693 | WB (1:1000) |
| antibody | anti-rabbit-AF647 (goat polyclonal) | ThermoFisher | A21244 | WB (1:4000) |
| antibody | anti-GAPDH (mouse monoclonal) | Abnova | clone 3C2 cat# H00002597-M01 | WB (1:2000) |
| antibody | anti-mouse IgG1-AF647 (goat polyclonal) | ThermoFisher | A21240 | FC (1µL per test), WB (1:4000) |
| antibody | HIV-Ig (human polyclonal) | Cummins, Weinhold et al. 1991; PMID 1995097 | NIH AIDS Reagent Program 3957 | WB (1:2000) |
| antibody | anti-human-AF647 (goat polyclonal) | ThermoFisher | A21445 | WB (1:4000) |
| antibody | anti-gp120 (sheep polyclonal) | Hatch, Tanaka et al. 1991; PMID 1374448 | NIH AIDS Reagent Program 288 | WB (1:1000) |
| antibody | anti-sheep-HRP (rabbit polyclonal) | Dako | P0163 | WB (1:20,000) |
| antibody | anti-gp41 (human monoclonal) | Zwick, Labrijn et al. 2001; PMID 11602729 | NIH AIDS Reagent Program 11557 | WB (1:1000) |
| antibody | anti-human (goat polyclonal) | ThermoFisher | 62-8420 | WB (1:10,000) |
| antibody | anti-Nef (rabbit polyclonal) | Shugars, Smith et al, 1993; PMID 8043040 | NIH AIDS Reagent Program 2949 | WB (1:1000) |
| antibody | anti-Vpr (rabbit polyclonal) | Dr. Jeffrey Kopp | NIH AIDS Reagent Program 11836 | WB (1:1000) |
| antibody | anti-rabbit (goat polyclonal) | ThermoFisher | 65-6120 | WB (1:10,000) |
| antibody | anti-GFP (chicken polyclonal) | Abcam | ab13970 | WB (1:1000) |
| antibody | anti-chicken-HRP (goat polyclonal) | ThermoFisher | A16054 | WB (1:10,000) |
| antibody | anti-STING (rabbit monoclonal) | Cell Signaling Technology | clone D2P2F cat# 13647 | WB (1:500) |
| antibody | anti-GBP5 (goat polyclonal) | Dr. Frank Kicrhhoff | sc-160353 | WB (1:500) |
| antibody | anti-IFITM3 (rabbit polyclonal) | Proteintech | 11714-1-AP | WB (1:1000) |
| antibody | anti-Env 2G12 (human monoclonal) | Buchacher, Predl et al. 1994; PMID 7520721 | NIH AIDS Reagent Program 1476 | neutralization (1µg/mL) |
| software, algorithm | FlowJo 10 | BD | 10.6.1 |  |
| software, algorithm | ABI Sequence Detection Software | ThermoFisher | 1.4 |  |
| software, algorithm | ImageQuant TL | GE | 8.2.0 |  |
| software, algorithm | Photoshop CC | Adobe | 20.0.6 |  |
| software, algorithm | shRNA retriever | http://katahdin.mssm.edu/siRNA/RNAi.cgi?type=shRNA |  |  |
